# Das BRCA-like-Mammakarzinom profitiert von Cisplatin plus Veliparib; Ergebnisse der S1416-Phase-II-Studie

**DOI:** 10.1007/s00066-023-02155-w

**Published:** 2023-10-12

**Authors:** Katharina Hintelmann, Kerstin Borgmann

**Affiliations:** 1 Ambulanzzentrum der UKE GmbH, Fachbereich Strahlentherapie, Universitätsklinikum Hamburg-Eppendorf, Martinistr. 52, 20246 Hamburg, Deutschland; 2 Labor für Strahlenbiologie & Experimentelle Radioonkologie, Klinik und Poliklinik für Strahlentherapie und Radioonkologie, Onkologisches Zentrum, Universitätsklinikum Hamburg-Eppendorf, Martinistr. 52, 20246 Hamburg, Deutschland

## Originalarbeit

Rodler E, Sharma P, Barlow WE et al (2023) Cisplatin with veliparib or placebo in metastatic triple-negative breast cancer and *BRCA* mutation-associated breast cancer (S1416): a randomised, double-blind, placebo-controlled, phase 2 trail. 24(2):162-174. 10.1016/S1470-2045(22)00739-2.

## Hintergrund

*BRCA1* und *BRCA2* sind die zentralen Gene der Reparatur der Desoxyribonukleinsäure (DNA) mittels homologer Rekombination (HR) und ihre Mutation wird in ca. 10–20 % des triple-negativen Brustkrebses (TNBC) beobachtet [1, 2]. Die Poly(ADP-Ribose)-Polymerase 1 (PARP1) ist an den Reparaturwegen „Basenexzisionsreparatur“ (BER; [3]) und „alternatives nichthomologes endjoining“ (altNHEJ; [4]) beteiligt, sodass eine PARP-Inhibition die Bildung von DNA-Schäden begünstigt. In Anlehnung an das Prinzip der „synthetic lethality“ sind insbesondere Tumoren mit einer defizienten HR vulnerabel für eine PARP-Inhibition [5] und es bietet sich eine Kombinationstherapie mit DNA-schädigenden Zytostatika oder Bestrahlung an. Bisherige Studien zeigten ein verbessertes progressionsfreies Überleben durch PARP-Inhibition verglichen mit Chemotherapie bei Brustkrebs mit vorliegender *BRCA1*-Keimbahnmutation. Die PARP1-Inhibitoren Olaparib und Talazoparib sind für dieses Kollektiv zugelassen. Neben der *BRCA*-Keimbahnmutation sind weitere Mechanismen bekannt, die zu einem Defizit in der HR (HRD) führen können. Studien zeigen in 40–60 % des TNBC ein HRD, was auch als BRCA-like-Phänotyp bezeichnet wird. Bisher liegen nur wenige Daten bezüglich der Wirksamkeit einer PARP-Inhibition für dieses Kollektiv vor. Das Ziel der S1416-Studie war, die Wirksamkeit des PARP-Inhibitors Veliparib in Kombination mit Cisplatin in metastasierten TNBC mit einem BRCA-like-Phänotyp im Vergleich zu *BRCA*-mutierten und *BRCA*-profizienten Tumoren zu untersuchen.

## Patienten und Methoden

SL1416 war eine randomisierte, doppelt verblindete und placebokontrollierte Phase-II-Studie mit 154 teilnehmenden Zentren in den USA. Eingeschlossen wurden Patientinnen mit einem metastasierten/rezidivierten TNBC oder einem mit einer Keimbahnmutation im *BRCA1*-Gen oder *BRCA2*-Gen (*BRCA1/2*) assoziierten, metastasierten/rezidivierten Brustkrebs und maximal einer vorherigen Chemotherapie in der metastasierten Situation. Die Patientinnen wurden 1:1 randomisiert und erhielten Cisplatin dreiwöchentlich (75 mg/m^2^ Körperoberfläche an Tag 1) und entweder Veliparib (300 mg, 2‑mal täglich, an den Tagen 1–14 des dreiwöchigen Zyklus) oder ein entsprechendes Placebo.

Als primärer Endpunkt wurde das progressionsfreie Überleben (PFS) erfasst. Zusätzlich zu den *BRCA1/2*-Mutationen wurden die Patientinnen anhand eines zuvor etablierten Biomarker-Panels in einen BRCA-like-Phänotyp subklassifiziert. Dieser BRCA-like Phänotyp wurde bei Vorliegen mindestens einer der folgenden Merkmale verwendet: I) Erhöhte genomische Instabilität (Wert für GI$\geq$42); II) somatische Mutation in *BRCA1/2*; III) Keimbahnmutation in HR-assoziierten Genen (*ATM, ATR, BABAM1, BAP1, BARD1, BLM, BRCA1, BRCA2 [FANCD1], BRIP1 [FANCJ], BRCC3, BRE, CHEK1, CHEK2, ERCC1, ERCC4 [FANCQ], FAM175A [Abraxas], FANCA, FANCB, FANCC, FANCD2, FANCE, FANCF, FANCG [XRCCC9], FANCI, FANCL, FAMCM, GEN1, MRE11A, NBN, PALB2 [FANCO], RAD50, RAD51C [FANCO], RAD51D, RBBP8 [CtIP], SLX4 [FANCP], UIMC1 [RAP80], XRCC2, TP53, PTEN, PI3KCA*) und IV) Methylierung des *BRCA1*-Promoters.

## Ergebnisse

Eingeschlossen wurden 335 Patientinnen zwischen Juli 2016 und Juni 2019. 247 Patientinnen wurden anhand vorliegender Biomarker einer der drei Subgruppen zugeordnet: *BRCA1/2*-Keimbahnmutation (*n* = 37), BRCA-like-Phänotyp (*n* = 101) und nicht „BRCA-like“ (*n* = 109). Die mittlere Nachbeobachtungszeit war 11,1 Monate. In der Gruppe mit *BRCA1/2*-Keimbahnmutation war das mittlere progressionsfreie Überleben 6,2 Monate unter Cisplatin/Veliparib und 6,4 Monate unter Cisplatin/Placebo (HR 0,79 [95 %-KI 0,38–1,67]; *p* = 0,54). In der Gruppe des BRCA-like-Phänotyps war das mittlere progressionsfreie Überleben 5,9 Monate unter Cisplatin/Veliparib gegenüber 4,2 Monate unter Cisplatin/Placebo (HR 0,57 [95 %-KI 0,37–0,88]; *p* = 0,01). In der Subgruppe ohne *BRCA*-Veränderungen war das mittlere progressionsfreie Überleben 4,0 Monate unter Cisplatin/Veliparib und 3 Monate unter Cisplatin/Placebo (HR 0,89 [95 %-KI 0,60–1,33]; *p* = 0,57).

Die häufigste therapieassoziierte Nebenwirkung von Grad 3 oder höher war eine Myelotoxizität, welche häufiger unter Cisplatin plus Veliparib auftrat als unter Cisplatin plus Placebo. Schwere therapieassoziierte Nebenwirkungen traten bei 31 % der Patienten unter Cisplatin plus Veliparib auf und bei 36 % der Patienten unter Cisplatin plus Placebo. In beiden Therapiearmen gab es einen therapieassoziierten Todesfall.

## Schlussfolgerung der Autoren

S1416 ist die erste Studie, in der die Wirksamkeit eines PARP-Inhibitors in Kombination mit einer platinhaltigen Chemotherapie bei metastasiertem Mammakarzinom mit Keimbahn-*BRCA1/2*-Wildtyp im Vergleich zu einem BRCA-like-Phänotyp untersucht wurde. Die höhere Wirksamkeit von Veliparib in der Gruppe der Patientinnen mit einem BRCA-like-Phänotyp eröffnet die Grundlage für den über die Keimbahn-*BRCA1/2*-Mutation hinausgehenden Einsatz von PARP-Inhibitoren. Weitere PARP-Inhibitoren könnten für die Kombination mit einer Platinchemotherapie in Betracht gezogen werden. Die Kombination von PARP-Inhibitoren mit einer Platinchemotherapie sollte deshalb in größeren randomisierten Studien für Patientinnen mit einem BRCA-like-Mammakarzinom weiter untersucht werden.

## Kommentar

Dies ist die erste Studie, die einen positiven Effekt der PARP-Inhibition in Kombination mit platinhaltiger Chemotherapie für Brustkrebs mit einem BRCA-like-Phänotyp für das PFS zeigt. Bisherige Studien in diesem Kontext fokussierten sich auf Patientinnen mit einer *BRCA1/2*-Keimbahnmutation und konnten einen Vorteil durch PARP-Inhibition für dieses Kollektiv zeigen. Erstaunlicherweise zeigte die S1416-Studie in dieser Gruppe keinen signifikanten Effekt. Dies könnte an der geringen Anzahl von 37 Patientinnen in diesem Arm liegen. Somit bleiben die Ergebnisse für *BRCA*-Mutations-Trägerinnen hinter den bisherigen Daten von Han et al. aus 2018 und Dieras et al. aus 2020 zurück. In diesen Arbeiten wurden allerdings Carboplatin und Paclitaxel mit PARP-Inhibition kombiniert. In einer Phase-II-Studie bei Patientinnen mit einem metastasierten/rezidivierten Brustkrebs und *BRCA*-Keimbahnmutation berichteten Han et al. einen signifikanten Vorteil für die Gesamtansprechrate und einen positiven Trend für das Gesamtüberleben (OS) und PFS für Veliparib in Kombination mit Carboplatin und Paclitaxel, verglichen mit Carboplatin und Paclitaxel allein [6]. Die BROCADE3-Phase-III-Studie zeigte bei fortgeschrittenem Brustkrebs mit *BRCA*-Keimbahnmutation eine Verlängerung des PFS für die Kombination Veliparib mit Carboplatin und Paclitaxel gegenüber Carboplatin und Paclitaxel. Es zeigte sich, dass das mediane PFS 14,5 Monate in der Veliparibgruppe gegenüber 12,6 Monaten in der Kontrollgruppe betrug (Hazard Ratio 0,71 [95 %-KI 0,57–0,88]; *p* = 0,0016) und keine deutlich erhöhten Nebenwirkungen in der Kombination mit Veliparib bestanden [7]. Bezüglich der PARP-Monotherapie gibt es ebenfalls überwiegend Daten für *BRCA*-Keimbahnmutations-Trägerinnen. Hier zeigten die Studien sowohl einen Vorteil für das PFS als auch für das OS [8–11]. 2020 veröffentlichten Tung et al. die Olaparib-Extended-Phase-II-Studie und zeigten ein Ansprechen auf eine Olaparibmonotherapie bei Brustkrebs mit einer somatischen *BRCA1/2*-Mutation oder einer *PALB2*-Keimbahnmutation. Bei einer somatischen *ATM*- oder *CHEK2*-Mutation allein wurde kein Ansprechen beobachtet [11, 12].

Zusammenfassend zeigt sich eine heterogene Landschaft bezüglich der untersuchten Mutationen, wohl auch bedingt durch den Einsatz unterschiedlicher PARP-Inhibitoren und Kombinationspartner; dies bestätigt Beobachtungen bei anderen Tumorentitäten. Beim kastrationsresistenten Prostatakarzinom schloss die PROpel-Studie Patienten unabhängig von ihrem HR-Status ein und zeigte in einer ersten Analyse die Verlängerung des PFS durch Olaparib in Kombination mit Abirateron und Prednisolon verglichen mit Abirateron und Prednisolon. Diskutiert wird eine Verringerung der HR-Kapazität durch den Kombinationspartner Abirateron und somit eine Wirksamkeit der PARP-Inhibition unabhängig vom HR-Status [13]. Auch die TOPARP-B-Studie konnte für die Olaparibmonotherapie eine Antitumoraktivität im metastasierten Prostatakarzinom, bei somatischer oder Keimbahnmutation in *BRCA1/2* oder bei Mutationen der HR-assoziierten Gene *ATM, PALB2, FANCA* und *CHEK2* zeigen [14]. Auch beim Ovarialkarzinom besteht eine Zulassung für den PARP-Inhibitor Niraparib unabhängig von molekularen Markern als Erhaltungstherapie nach einem Ansprechen auf eine platinhaltige Chemotherapie sowie die Zulassung für Olaparib für die selbige Situation neben der *BRCA1/2*-Keimbahnmutation auch für eine somatische *BRCA1/2*-Mutation.

Insgesamt sprechen die Daten weiterhin für die Relevanz des HR-Status für ein Ansprechen auf eine PARP-Inhibition. Da die Therapie im Allgemeinen gut toleriert wird und mit unterschiedlichsten Kombinationstherapien eingesetzt wurde, ist dies eine vielversprechende Option für das Mammakarzinom. Der „benefit“ jenseits der *BRCA1/2*-Keimbahnmutation sollte somit dringend weiter untersucht werden.

Interessant ist, dass in der S1416-Studie insbesondere für Patientinnen ein Vorteil gesehen wurde, die zuletzt keine vorhergehende Therapielinie hatten, also nicht unter der letzten Therapie refraktär/progredient waren. Dies zeigt Ähnlichkeit zu den Daten des Ovarialkarzinoms, bei dem die PARP-Inhibition platinresponsiver Tumoren ebenfalls effektiv ist. Dies weist auf die Wirksamkeit beider Therapeutika insbesondere in proliferierenden Tumoren hin.

Ein weiterer wichtiger Aspekt ist der mögliche Einsatz von PARP-Inhibitoren als Radiosensitizer, wofür zahlreiche positive präklinische Daten vorliegen, beispielhaft dargestellt in [15]. Durch die induzierten DNA-Schäden nach ionisierender Bestrahlung sind Zellen mit einem HRD in einer größeren Abhängigkeit durch die PARP-vermittelte DNA-Reparatur, sodass eine Verstärkung des Antitumoreffekts durch PARP-Inhibition zu erwarten ist. Die RADIOPARP-Phase-I-Studie zeigte hier immerhin schon eine gute Tolerabilität beim TNBC [16].

Man sollte für das Design zukünftiger Studien jedoch noch stärker die Verwendung geeigneter Biomarker bedenken und die bestmögliche Auswahl des Patientinnenkollektivs berücksichtigen. Es ist sicherlich ein sinnvoller Ansatz, neben Keimbahnmutationen in *BRCA1* und *BRCA2* auch den HRD-Score in die Vorhersage eines Ansprechens auf PARP-Inhibitoren einzubeziehen. Dieser über die simple Analyse der beiden Gene hinausgehende Score berücksichtigt neben einzelnen Keimbahnmutationen in den *BRCA1/2*-Genen auch den Verlust der Heterozygotie, die allelische Imbalance der Telomere und die Transitionen längerer Abschnitte von genomischer DNA, wobei jedes Merkmal für sich allein bereits als Marker einer genomischen Instabilität betrachtet werden kann [17–24]. Dieser auch als 3‑Biomarker-Signatur bezeichnete Assay erlangte bereits die FDA-Zulassung für die Bestimmung des HRD, mit einem GI-Wert größer als 42 beim Ovarialkarzinom [22]. Allerdings erfordert diese Diagnose, dass Tumorproben mit *„next generation sequencing“* analysiert werden, also einen Ansatz, der im klinischen Alltag möglicherweise nicht trivial durchführbar ist.

Darüber hinaus ist verwirrend, dass der Begriff HRD-Score in der Community sehr dehnbar ausgelegt wird und bei der Suche in PubMed zu aktuell 141 Publikationen führt, die nicht nur den wie oben beschriebenen HRD-Score benennen, sondern dessen individuelle Varianten. Diese müssten ebenfalls in Studien bezüglich ihres Aussagewerts überprüft werden. Einige Studien analysieren direkt die Funktionalität der HR hinsichtlich ihrer sichtbaren Aktivität und könnten dadurch nicht nur leichter durchführbar sein, sondern auch ein generelles Bild der HR widerspiegeln. Es wäre in diesem Zusammenhang noch stärker zu berücksichtigen, dass eine *BRCA1*-Mutation nicht automatisch zu einem Verlust der Funktion führen muss. Zu den vielversprechenden Ansätzen der Funktionalität gehört der Nachweis von RAD51. RAD51 ist das Schlüsselprotein der HR, welches lokalisiert am DNA-Schaden akkumuliert und als sogenannter Reparaturfokus nachgewiesen werden kann. Die Bildung eines solchen Fokus wird als „read-out“ für eine funktionelle HR angesehen. Es zeigte sich, dass in BRCA-like-Tumoren kein RAD51 in Tumorgewebe der entsprechenden Patientinnen nachweisbar war und eine PARP-Inhibition für diese Tumoren vielversprechend sein könnte [25, 26].

### Fazit

Dies ist die erste Studie, die einen positiven Effekt der PARP-Inhibition in BRCA-like-Tumoren für das progressionsfreie Überleben in Kombination mit Chemotherapie zeigt. Bisherige Studien fokussierten sich auf die Analyse von Patientinnen mit *BRCA1/2*-Keimbahnmutationen und konnten auch für diese einen Vorteil in der mit PARP-Inhibitoren behandelten Kohorte beobachten. Gerade vor dem Hintergrund von etwa 40–60 % BRCA-like-Mammakarzinomen wäre die Ausweitung der Anwendung von PARP-Inhibitoren auf diese Patientinnenkohorte wünschenswert. Eine sorgfältige Auswahl der Patientinnen durch eine funktionelle Testung könnte die Selektion von Patientinnen mit der höchsten Ansprechwahrscheinlichkeit positiv unterstützen.

*Katharina Hintelmann und Kerstin Borgmann, Hamburg*

## References

[1] Sharma P (2014). Germline BRCA mutation evaluation in a prospective triple-negative breast cancer registry: implications for hereditary breast and/or ovarian cancer syndrome testing. Breast Cancer Res Treat.

[2] Couch FJ (2015). Inherited mutations in 17 breast cancer susceptibility genes among a large triple-negative breast cancer cohort unselected for family history of breast cancer. J Clin Oncol.

[3] Ronson GE (2018). PARP1 and PARP2 stabilise replication forks at base excision repair intermediates through Fbh1-dependent Rad51 regulation. Nat Commun.

[4] Audebert M, Salles B, Calsou P (2004). Involvement of poly(ADP-ribose) polymerase-1 and XRCC1/DNA ligase III in an alternative route for DNA double-strand breaks rejoining. J Biol Chem.

[5] Bryant HE (2005). Specific killing of BRCA2-deficient tumours with inhibitors of poly(ADP-ribose) polymerase. Nature.

[6] Han HS (2018). Veliparib with temozolomide or carboplatin/paclitaxel versus placebo with carboplatin/paclitaxel in patients with BRCA1/2 locally recurrent/metastatic breast cancer: randomized phase II study. Ann Oncol.

[7] Dieras V (2020). Veliparib with carboplatin and paclitaxel in BRCA-mutated advanced breast cancer (BROCADE3): a randomised, double-blind, placebo-controlled, phase 3 trial. Lancet Oncol.

[8] Tung NM (2020). TBCRC 048: Phase II Study of Olaparib for Metastatic Breast Cancer and Mutations in Homologous Recombination-Related Genes. J Clin Oncol.

[9] Geyer CE (2022). Overall survival in the OlympiA phase III trial of adjuvant olaparib in patients with germline pathogenic variants in BRCA1/2 and high-risk, early breast cancer. Ann Oncol.

[10] Tutt ANJ (2021). Adjuvant Olaparib for Patients with BRCA1- or BRCA2-Mutated Breast Cancer. N Engl J Med.

[11] Litton JK (2018). Talazoparib in Patients with Advanced Breast Cancer and a Germline BRCA Mutation. N Engl J Med.

[12] Tung NM (2020). Management of Hereditary Breast Cancer: American Society of Clinical Oncology, American Society for Radiation Oncology, and Society of Surgical Oncology Guideline. J Clin Oncol.

[13] Saad F (2022). Patient-reported outcomes with olaparib plus abiraterone versus placebo plus abiraterone for metastatic castration-resistant prostate cancer: a randomised, double-blind, phase 2 trial. Lancet Oncol.

[14] Mateo J (2020). Olaparib in patients with metastatic castration-resistant prostate cancer with DNA repair gene aberrations (TOPARP-B): a multicentre, open-label, randomised, phase 2 trial. Lancet Oncol.

[15] Michmerhuizen AR (2019). PARP1 Inhibition Radiosensitizes Models of Inflammatory Breast Cancer to Ionizing Radiation. Mol Cancer Ther.

[16] Loap P (2021). Combination of Olaparib and Radiation Therapy for Triple Negative Breast Cancer: Preliminary Results of the RADIOPARP Phase 1 Trial. Int J Radiat Oncol Biol Phys.

[17] Abkevich V (2012). Patterns of genomic loss of heterozygosity predict homologous recombination repair defects in epithelial ovarian cancer. Br J Cancer.

[18] Birkbak NJ (2012). Telomeric allelic imbalance indicates defective DNA repair and sensitivity to DNA-damaging agents. Cancer Discov.

[19] Popova T (2012). Ploidy and large-scale genomic instability consistently identify basal-like breast carcinomas with BRCA1/2 inactivation. Cancer Res.

[20] Timms KM (2014). Association of BRCA1/2 defects with genomic Scores predictive of DNA damage repair deficiency among breast cancer subtypes. Breast Cancer Res.

[21] Telli ML (2015). Phase II Study of Gemcitabine, Carboplatin, and Iniparib As Neoadjuvant Therapy for Triple-Negative and BRCA1/2 Mutation-Associated Breast Cancer With Assessment of a Tumor-Based Measure of Genomic Instability: PrECOG 0105. J Clin Oncol.

[22] Telli ML (2016). Homologous Recombination Deficiency (HRD) Score Predicts Response to Platinum-Containing Neoadjuvant Chemotherapy in Patients with Triple-Negative Breast Cancer. Clin Cancer Res.

[23] Telli ML (2018). Homologous recombination deficiency (HRD) status predicts response to standard neoadjuvant chemotherapy in patients with triple-negative or BRCA1/2 mutation-associated breast cancer. Breast Cancer Res Treat.

[24] Lenz L et al (2023) Identifying homologous recombination deficiency in breast cancer: genomic instability Score distributions differ among breast cancer subtypes. Breast Cancer Res Treat 202(1):191–201. 10.1007/s10549-023-07046-3 10.1007/s10549-023-07046-3 PMC10504389 37589839

[25] Naipal KA (2014). Functional ex vivo assay to select homologous recombination-deficient breast tumors for PARP inhibitor treatment. Clin Cancer Res.

[26] Kramer CJ et al (2023) RAD51 as a biomarker for homologous recombination deficiency in high-grade serous ovarian carcinoma: robustness and interobserver variability of the RAD51 test. J Pathol Clin Res 9(6):442–448. 10.1002/cjp2.336. 10.1002/cjp2.336 PMC10556259 37504067

